# Eicosapentaenoic and docosahexaenoic acid-enriched high fat diet delays the development of fatty liver in mice

**DOI:** 10.1186/s12944-015-0072-8

**Published:** 2015-07-22

**Authors:** Nikul K Soni, Intawat Nookaew, Ann-Sofie Sandberg, Britt G Gabrielsson

**Affiliations:** Divisions of Food and Nutrition Science, Department of Biology and Biological Engineering, Chalmers University of Technology, SE-41296 Gothenburg, Sweden; The division of Systems and Synthetic Biology, Department of Biology and Biological Engineering, Chalmers University of Technology, SE-41296 Gothenburg, Sweden; Present address: Comparative Genomics Group, Biosciences division, Oak Ridge National Library, Oak Ridge, TN 37831 USA

**Keywords:** Diet, Liver triglycerides, Transcriptome, Lipid droplets, Ppar

## Abstract

**Background:**

Low hepatic content of n-3 PUFA has been associated with NAFLD in humans. Whether this is associated with reduced dietary intake or increased turnover of these FA is not clear. We have here investigated the effects of dietary fat quality on hepatic lipid storage and transcriptomics over time.

**Aim:**

To investigate the effects of quality of fat in a high fat diet (HFD) over time on hepatic lipid storage and liver transcriptomics.

**Methods and Results:**

Male C57BL/6J mice were fed control, HFD-eicosapentaenoic acid (EPA)/ docosahexaenoic acid (DHA) or HFD-corn oil diet for 8 or 12 weeks. Body weight, body composition, plasma and hepatic triglyceride contents were measured. Hepatic transcriptomes were analysed by microarray followed by gene-set enrichment analyses. At 8 weeks, the HFD-corn oil mice had higher body weight and adipose depot mass than the HFD-EPA/DHA but there were no differences at 12 weeks. Hepatic triglyceride content was lower in HFD-EPA/DHA fed compared with the HFD-corn oil fed mice at both time-points. Enrichment analyses of the hepatic transcriptomes showed that lipid/fatty acid biosynthesis; transport and homeostasis were lower in the HFD-EPA/DHA fed compared with the HFD-corn oil fed mice. Genes encoding proteins associated to cytoplasmic lipid droplets were expressed at higher levels in livers from the HFD-corn oil compared to HFD-EPA/DHA mice.

**Conclusions:**

Dietary EPA and DHA counteracted development of HFD-induced fatty liver. The liver transcriptome data implicate that the quality of dietary fat could modulate Ppar-related gene expression that in turn affects hepatic lipid storage and maintenance of metabolic health.

**Electronic supplementary material:**

The online version of this article (doi:10.1186/s12944-015-0072-8) contains supplementary material, which is available to authorized users.

## Introduction

It is becoming more established that an increased lipid content in the liver can be viewed as a driver of the metabolic dysfunctions that are observed in obesity [1]. Non-alcoholic fatty liver disease (NAFLD) covers the spectrum of liver damage from simple fatty liver to steatohepatitis and cirrhosis [2]. About 70 % of subjects with type 2 diabetes or the metabolic syndrome develop NAFLD [3]. Further, a recent retrospective study showed that subjects diagnosed with NAFLD or non-alcoholic steatohepatitis (NASH) had reduced life expectancy due to an increased mortality of 69 % and 86 %, respectively, primarily caused by cardiovascular disease [4].

It has been suggested that a high dietary intake of sucrose/fructose, cholesterol and saturated fat can be associated with NAFLD in humans [5, 6]. Whether reduced dietary intake of n-3 fatty acids is related to NAFLD progression, and whether low hepatic n-3 levels are cause or consequence of NASH is not clear. In this study, groups of male C57BL/6J mice were fed either a control diet or two almost identical high-fat diets (HFDs; 31.7 E% fat) with only minor differences in fat quality. One was based on corn oil (HFD-corn oil) and the other was enriched in the n-3 PUFA eicosapentaenoic acid (EPA) and docosahexaenoic acid (DHA; HFD-EPA/DHA). The amount of EPA and DHA included in the diet was calculated from a previous study where we used a herring-based diet [7, 8]. The two HFDs had cholesterol added since previous research has shown that the combination of high fat and cholesterol in the diet is required to induce fatty liver in mice [9].

The aims of this study were to investigate the effects of dietary EPA and DHA *vs.* corn oil in a HFD on hepatic triglyceride storage in liver over time and to identify putative mechanisms of action by transcriptomics.

## Results

### Diets

With the exception of fat sources, all diets were formulated from the same sources of macro- and micronutrients enabling the direct comparison of the different fat qualities as the main effector across the diets. The control diet contained 12 E% fat and the HFDs contained 32 E% fat. The two HFDs differed as follows: the HFD-corn oil contained 11 E% of corn oil, with high linoleic acid content, and the HFD-EPA/DHA diet contained 6 E% corn oil and 4 E% EPA/DHA-enriched oils (Table 1). The content of EPA plus DHA in the HFD-EPA/DHA diet was 8 g/kg and was based on a herring-based diet we previously have used [7, 8]. The amount of protein was increased in the HFDs to achieve similar E%. The amount of sugar and corn starch was adjusted to accommodate the higher fat content in the HFDs.

**Table 1:** Compositions of control, high fat diet (HFD)- EPA/DHA (ED) and HFD-corn oil (CO)

Ingredient (g/100 g diet)		Control	HFD ED	HFD CO
Protein	Casein	22.2	25.6	25.6
Carbohydrates	Sucrose	5.0	10.0	10.0
	Corn starch	56.0	34.8	34.8
	Cellulose	5.0	5.8	5.8
Fat	Total	5.0	15.0	15.0
	Corn oil	2.5	3.0	5.0
	Coconut oil	2.5	10.0	10.0
	EPAX oils^a^	0	2.0	0
Minerals^b^		2.0	2.5	2.5
Miconutrients^c^		3.0	3.0	3.0
Choline bitartrate		1.6	2.0	2.0
Cholesterol		0	1.0	1.0
Methionine		0.2	0.3	0.3
Energy content (kJ/100 g)		1599	1752	1752
	Protein E%	24	25	25
	Carbohydrate E%	65	44	44
	Fat E%	12	32	32
Fatty acid composition^d^ (mg/g diet)				
	C10:0	0.20	1.47	1.33
	C12:0	2.37	7.58	7.72
	C14:0	1.54	4.58	4.78
	C16:0	1.90	3.44	3.59
	C18:0	0.68	2.26	2.49
	C18:1n-9	2.82	4.80	5.26
	C18:2n-6	3.62	5.03	7.36
	C18:3n-6	0.12	0.22	0.26
	C20:5n-3 (EPA)	0.00	2.03	0.01
	C22:6n-3 (DHA)	0.00	4.58	0.01
	Sum SFA	6.70	19.33	19.91
	Sum MUFA	2.82	4.80	5.26
	Sum n-6 PUFA	3.74	5.26	7.62
	Sum n-3 PUFA	0.00	6.61	0.02

^a^EPAX 1050, EPAX 6015

^b^CaCO_3_ (57.7 %); KCl (19.9 %); KH_2_PO_4_ (11.9 %); MgSO_4_ (10.4 %)

^c^Corn starch (98.22 %); Ca(IO_3_)_2_ (0.0007 %); CoCO_3_ (0.064 %); CuO (0.02 %); FeSO_4_ (0.5 %); MnO_2_ (0.035 %); Na_2_MoO_4_ (0.001 %); NaSeO_3_ (0.0007 %); ZnO (0.1 %); Vitamin A (0.013 %); B_2_ (Riboflavin-5-phosphate sodium; 0.027 %); B3 (0.1 %); B_5_ (Ca Pantothenate; 0.057 %); B_6_ (0.023 %); B_7_ (0.0007 %); B_9_ (0.007 %); B_12_ (0.00008 %); D_3_ (0.007 %); E (0.25 %); K (0.003 %)

^d^Analyses were performed in triplicates and data obtained by Gas chromatography mass spectroscopy

### Delayed body weight gain and adiposity in the HFD-EPA/DHA mice

**Fig. 1:**
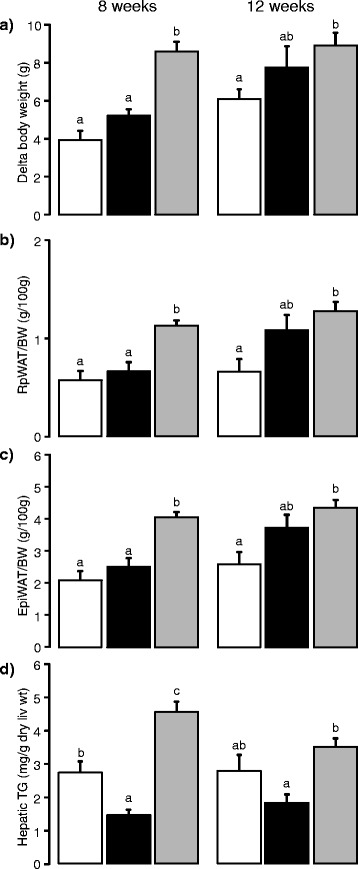
Effects of 8 weeks (left panel) or 12 weeks (right panel) diet with control diet (white bars). HFD-EPA/DHA (black bars) and HFD-corn oil (grey bars): **a**) body weight change. **b**) rpWAT. **c**) epiWAT. **d**) Hepatic triglyceride content. Data are shown as mean ± SEM; 8 weeks: n = 10. 12. 12; 12 weeks: n = 11. 12. 12; control. HFD-EPA/DHA. HFD-corn oil; respectively. Different letters (e.g. a, b, c) denote significant difference of p < 0.05 between groups as evaluated by the Tukey’s multiple comparison test (see further the Methods section)

At 8 weeks, the HFD-corn oil mice had higher body weight gain compared to both the control and the HFD-EPA/DHA mice but there was no difference between HFD-EPA/DHA and the control mice (Fig. 1a left panel). However, the relative retroperitoneal white adipose tissue (rpWAT) and epididymal white adipose tissue (epiWAT) weights were higher in the HFD-corn oil mice compared to both the control and to the HFD-EPA/DHA mice (Fig. 1b and c, left panel). There were no differences in the relative rpWAT and epiWAT weights between HFD-EPA/DHA and the control mice.

At 12 weeks, there were no differences in body weight gain between the HFD-EPA/DHA mice compared to mice fed the other two diets (Fig. 1a, right panel). The HFD-corn oil mice had higher body weight change than the controls. The body fat, measured by Dual energy X-ray absorptiometry (DXA), was higher in the HFD-corn oil mice (30.6 ± 1.6 body fat %) compared with the controls (19.3 ± 1.5 %; p = 0.001). The HFD-EPA/DHA mice (26.3 ± 2.4 body fat %) did not differ from either the control or the HFD-corn oil mice. In agreement with the DXA, the relative rpWAT and epiWAT weights were higher in the HFD-corn oil mice compared with the controls (Fig. 1b and c, right panels). The relative rpWAT or epiWAT weights from the HFD-EPA/DHA mice not differ from either the control or the HFD-corn oil mice.

### Reduced hepatic triglyceride content in the HFD-EPA/DHA mice

There were no effects on the relative liver weights (g/100 g body weight) after the 8-week diet intervention (Table 2). At 12 weeks, the relative liver weights were lower in the HFD-EPA/DHA mice compared with the HFD-corn oil mice whereas the control relative liver weights did not differ from either HFD.

**Table 2:** Dietary effects on liver weight, plasma and hepatic lipid content

Parameter	8 weeks			12 weeks		
	Control	HFD ED	HFD CO	Control	HFD ED	HFD CO
n	9	12	12	10	9	10
Liver (% Bw)	4.4 ± 0.5	4.5 ± 0.3	4.2 ± 0.2	4.6 ± 0.1^ab^	4.3 ± 0.1^a^	4.8 ± 0.1^b^
Hepatic total lipid (mg/g dry liver weight)^a^	4.6 ± 0.3^a^	3.9 ± 0.2^a^	6.9 ± 0.4^b^	5.2 ± 0.5^ab^	4.7 ± 0.3^a^	6.5 ± 0.5^b^
Plasma triglycerides (mmole/L)	0.9 ± 0.1^ab^	1.1 ± 0.1^a^	0.7 ± 0.0^b^	1.2 ± 0.1	1.0 ± 0.1	1.0 ± 0.1
Hepatic total cholesterol (mg/g dry liver weight)	5.0 ± 0.2^a^	6.2 ± 0.4^b^	6.0 ± 0.1^b^	4.4 ± 0.1^a^	6.4 ± 0.2^b^	6.4 ± 0.2^b^
Plasma total cholesterol (mmole/L)	5.6 ± 0.9	5.5 ± 0.8	5.4 ± 0.9	6.3 ± 0.3^a^	6.0 ± 0.3^a^	9.2 ± 0.5^b^

Data are shown as mean ± SEM; different letters show significant differences tested by ANOVA followed by Tukey’s multiple comparison test

^a^Lipid measurements n = 7, 8, 8 for Control, HFD-EPA/DHA and HFD-corn oil, respectively. High fat diet (HFD)- EPA/DHA (ED) and HFD-corn oil (CO)

At 8 weeks, the HFD-corn oil mice had higher content of hepatic total lipids compared to both HFD-EPA/DHA and control mice (Table 2). At 12 weeks, the total lipid content remained lower in the livers from the HFD-EPA/DHA mice compared with the HFD-corn oil mouse. There were no differences between the controls and either HFD in total liver lipid content at 12 weeks. After the 8-week diet intervention, the hepatic triglyceride content in livers from the HFD-corn oil mice was approximately 1.7-fold higher than the control livers and 3-fold higher than the HFD-EPA/DHA livers (Fig. 1d, left panel). Furthermore, the triglyceride content of the HFD-EPA/DHA livers was about 0.5-fold of the control livers. The differences in hepatic triglyceride content in the HFD-corn oil and the HFD-EPA/DHA-fed mice remained significant after 12 weeks (Fig. 1d, right panel). The triglyceride content of the control livers did not differ from either of the two HFDs after the 12 weeks diet intervention. The cholesterol content in the liver did not differ between two HFDs but it was higher in both HFDs compared to control at 8 and 12 weeks diet intervention (Table 2).

The plasma triglyceride concentrations were higher in the HFD-EPA/DHA mice compared with the HFD-corn oil mice after 8 weeks (Table 2). Neither HFD group differed from the control plasma triglyceride levels. There were no differences in plasma triglyceride concentrations after 12 weeks of dietary intervention. There were no differences in plasma cholesterol concentrations after 8 weeks but at 12 weeks the plasma levels were higher in mice fed HFD-corn oil compared to HFD-EPA/DHA and control.

### Hepatic fatty acid composition

#### Neutral lipids

Table 3 shows amount of individual fatty acids of interest in the hepatic neutral lipid fraction. The levels of saturated fatty acid (SFA) C16:0 at 8 weeks were lower in livers from the control and the HFD-EPA/DHA fed mice compared to those fed HFD-corn oil. At 12 weeks, the amount of C16:0 remained lower in the HFD-EPA/DHA livers compared to the HFD-corn oil livers whereas the controls did not differ from either. There were no differences in the levels of C18:0 after 8 weeks diet intervention. The levels of C18:0 were lower in HFD-EPA/DHA fed mice compared to HFD-corn oil at 12 weeks. The MUFA content in livers from the HFD-corn oil mice were much higher than that found in the control or HFD-EPA/DHA livers at both 8 weeks and 12 weeks. As expected, the levels of EPA (C20:5n-3) and DHA (C22:6n-3) were higher in HFD-EPA/DHA fed mice at both time points.

**Table 3:** Liver fatty acids profiles

	8 week			12 week		
Neutral Lipids	Control	HFD ED	HFD CO	Control	HFD ED	HFD CO
Sum SFA	15.5 ± 1.3	12.3 ± 2.0	26.3 ± 1.7	20.6 ± 2.5	15.6 ± 1.8	23.6 ± 1.3
C16:0	13.5 ± 1.2^a^	10.2 ± 1.5^a^	23.9 ± 1.6^b^	18.2 ± 2.3^ab^	13.8 ± 1.6^a^	21.2 ± 1.2^b^
C18:0	2.1 ± 0.1	2.1 ± 0.5	2.4 ± 0.2	2.3 ± 0.2^ab^	1.9 ± 0.2^a^	2.4 ± 0.1^b^
Sum MUFA	28.0 ± 3.3	11.0 ± 2.8	44.3 ± 3.5	37.1 ± 6.3	19.9 ± 3.5	49.6 ± 4.0
C16:1n-7	4.1 ± 0.5^a^	2.2 ± 0.6^b^	1.8 ± 0.2^b^	5.5 ± 0.9^a^	4.2 ± 0.7^a^	9.6 ± 0.7^b^
C18:1n-9	19.4 ± 2.1^a^	7.9 ± 1.9^b^	33.9 ± 2.6^c^	25.1 ± 4.4^ab^	13.9 ± 2.4^a^	31.7 ± 2.6^b^
C18:1n-7	4.5 ± 0.7^a^	0.9 ± 0.3^b^	8.5 ± 0.8^c^	6.5 ± 1.1^a^	1.7 ± 0.4^b^	8.2 ± 0.7^a^
Sum n-6 PUFA	9.4 ± 0.4	7.7 ± 1.3	14.9 ± 0.8	8.9 ± 1.0	7.9 ± 0.5	17.1 ± 0.9
C18:2n-6	6.9 ± 0.3^a^	6.8 ± 1.0^a^	14.9 ± 0.8^b^	7.2 ± 0.7^a^	7.9 ± 0.5^a^	14.9 ± 0.7^b^
C20:4n-6	2.4 ± 0.1^a^	0.9 ± 0.3^b^	0.0 ± 0.0^c^	1.7 ± 0.2^a^	0.0 ± 0.0^b^	2.2 ± 0.3^a^
Sum n-3 PUFA	0.0 ± 0.0	9.2 ± 1.5	0.0 ± 0.0	0.3 ± 0.0	11.5 ± 1.3	0.0 ± 0.0
C20:5n-3 (EPA)	0.0 ± 0.0^a^	1.6 ± 0.3^b^	0.0 ± 0.0^a^	0.0 ± 0.0^a^	2.3 ± 0.3^b^	0.0 ± 0.0^a^
C22:6n-3 (DHA)	0.0 ± 0.0^a^	7.6 ± 1.2^b^	0.0 ± 0.0^a^	0.3 ± 0.0^a^	9.3 ± 1.0^b^	0.0 ± 0.0^a^
Phospholipids						
Sum SFA	19.4 ± 0.7	23.0 ± 1.2	17.1 ± 0.5	20.5 ± 0.5	21.7 ± 1.2	17.8 ± 1.0
C16:0	11.6 ± 0.4^a^	15.3 ± 0.7^b^	10.7 ± 0.3^a^	12.6 ± 0.3^ab^	14.7 ± 0.8^b^	11.2 ± 0.6^a^
C18:0	7.8 ± 0.3^a^	7.7 ± 0.5^a^	6.4 ± 0.2^b^	8.0 ± 0.2^a^	7.0 ± 0.4^ab^	6.6 ± 0.4^b^
Sum MUFA	7.2 ± 0.2	6.5 ± 0.4	6.40.2	7.5 ± 0.1	6.2 ± 0.3	6.3 ± 0.2
C16:1n-7	1.0 ± 0.0	0.9 ± 0.1	1.1 ± 0.1	0.9 ± 0.0	0.9 ± 0.1	1.0 ± 0.0
C18:1n-9	6.2 ± 0.2^a^	5.6 ± 0.3^ab^	5.3 ± 0.1^b^	6.6 ± 0.1^a^	5.4 ± 0.2^b^	5.3 ± 0.2^b^
Sum n-6 PUFA	21.2 ± 0.8	11.7 ± 1.1	19.8 ± 1.4	19.6 ± 0.8	11.7 ± 0.8	20.8 ± 0.9
C18:2n-6	6.0 ± 0.3	6.6 ± 1.0	6.2 ± 0.9	5.4 ± 0.2a	6.6 ± 0.5ab	7.7 ± 0.4b
C20:3n-6	1.2 ± 0.1^a^	0.4 ± 0.0^b^	2.0 ± 0.1^c^	1.2 ± 0.1^a^	0.6 ± 0.0^b^	1.7 ± 0.1^c^
C20:4n-6	13.9 ± 0.4^a^	4.7 ± 0.1^b^	11.6 ± 0.4^c^	13.0 ± 0.5^a^	4.6 ± 0.3^b^	11.3 ± 0.4^c^
Sum n-3 PUFA	3.5 ± 0.1	13.2 ± 2.0	2.8 ± 0.1	2.7 ± 0.2	12.4 ± 0.8	2.2 ± 0.1
C20:5n-3 (EPA)	0.0 ± 0.0^a^	0.4 ± 0.0^b^	0.0 ± 0.0^a^	0.0 ± 0.0^a^	0.4 ± 0.0^b^	0.0 ± 0.0^a^
C22:6n-3 (DHA)	3.5 ± 0.1^a^	12.7 ± 2.0^b^	2.8 ± 0.1^a^	2.7 ± 0.2^a^	12.4 ± 0.8^b^	2.2 ± 0.1^a^

Data are presented as mg/g dry liver weight and shown as mean±SEM. Different letter shows significant differences tested by ANOVA followed by Tukey's multiple comparison test

Liver fatty acid profiles n = 7, 8, 8 for Control, HFD ED and HFD CO, respectively. High fat diet (HFD)- EPA/DHA (ED) and HFD-corn oil (CO)

#### Phospholipids

Table 3 shows the fatty acid composition of the hepatic phospholipid fraction. At 8 weeks, the phospholipid C16:0 content was higher in livers from mice fed the HFD-EPA/DHA compared to mice fed the other two diets. Furthermore, the phospholipid C18:0 content was lower in HFD-corn oil compared to other two diets, whereas there was no difference in the C18:0 content between the control and the HFD-EPA/DHA fed mice. At 12 weeks higher amounts of C16:0 was present in HFD-EPA/DHA fed mice compared to HFD-corn oil but the amounts did not differ when compared to control. Also, the HFD-corn oil fed mice had lower amounts of C18:0 to control but did not differ from HFD-EPA/DHA. There were no difference in the C16:1n-7 levels at both time points whereas, the amounts of C18:1n-9 were lower in HFD-corn oil fed mice compared to control at 8 weeks. Also, C18:1n-9 levels were higher in control compared to HFD-EPA/DHA and HFD-corn oil at 8 weeks. There were no difference in C18:2n-6 content at 8 weeks but at 12 weeks higher levels was detected in HFD-corn oil fed mice compared to control. The levels of C20:4n-6 (arachidonic acid) was lower in HFD-EPA/DHA compared to either diet at both the time points whereas the levels of EPA and DHA were higher after the HFD-EPA/DHA feeding.

### Time-dependent effects of high fat diet on hepatic pathways

Additional file 1: Figure S4a shows that the Gene Ontology (GO) Biological Processes (BPs) that were upregulated by both HFDs *vs*. control at 8 weeks mainly related to cell cycle/DNA replication, fatty acid β-oxidation and protein transport. Examples of BPs downregulated were cholesterol, sterol or isoprenoid biosynthesis, and signalling via G-protein coupled receptors (GPCR). At 12 weeks, the BPs upregulated by both HFDs *vs.* control involved drug metabolic processes, fatty acid metabolism and positive regulation of sequestering of triglyceride (Additional file 1: Figure S4b). BPs downregulated were cholesterol, sterol, very long-chain fatty acid and lipid biosynthetic processes and GPCR signalling pathways.

### Time-dependent effects of dietary fat quality on hepatic pathways

**Fig. 2:**
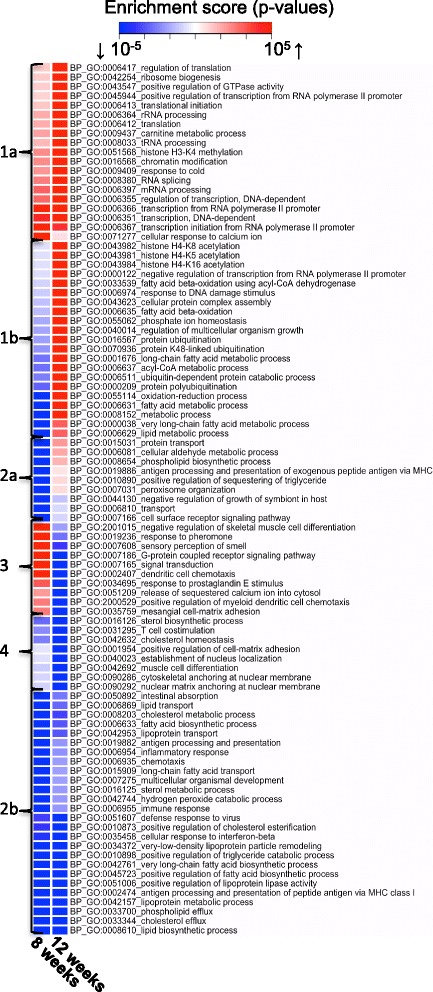
Heatmap of GO: BPs from the gene set enrichment analysis of transcriptome data from HFD-EPA/DHA *vs* HFD-corn oil comparisons at 8 weeks (left column) and 12 weeks (right column). Clusters 1a/b to 4 are shown and discussed in the text. The colour intensities show the level of enrichment of each GO:BP term where blue signifies downregulated processes and red upregulated; FDR adjusted p-value <0.05

Biological processes that were up- or downregulated by HFD-EPA/DHA *vs.* HFD-corn oil at 8 and at 12 weeks are shown in Fig. 2. The BPs making up cluster 1a and 1b showed little regulation at 8 weeks but were upregulated by HFD-EPA/DHA at 12 weeks. These clusters include processes like transcription (histone methylation/acetylation; chromatin modification), translation (mRNA/rRNA/tRNA processing, regulation of translation) and protein turnover (polyubiquitination). Two BPs related to fatty acid β-oxidation were also found here. Cluster 2a contained BPs downregulated by HFD-EPA/DHA *vs.* HFD-corn oil at week 8 but not at week 12. These BPs involved protein transport and metabolism of lipids and fatty acids. Cluster 2b at the bottom of the heatmap is composed of BPs downregulated by HFD-EPA/DHA *vs.* HFD-corn oil at both time points. These processes mainly involved lipid/sterol metabolism, lipid and fatty acid biosynthesis, lipoprotein transport and cholesterol/phospholipid efflux. Cluster 3 and 4 show BPs that were either not affected or upregulated by HFD-EPA/DHA *vs* HFD-corn oil at 8 weeks followed by downregulation at 12 weeks. Members of this cluster related to signaling particularly via GPCR, chemotaxis or adhesion.

### Time-dependent effects of diet on gene expression

**Fig. 3:**
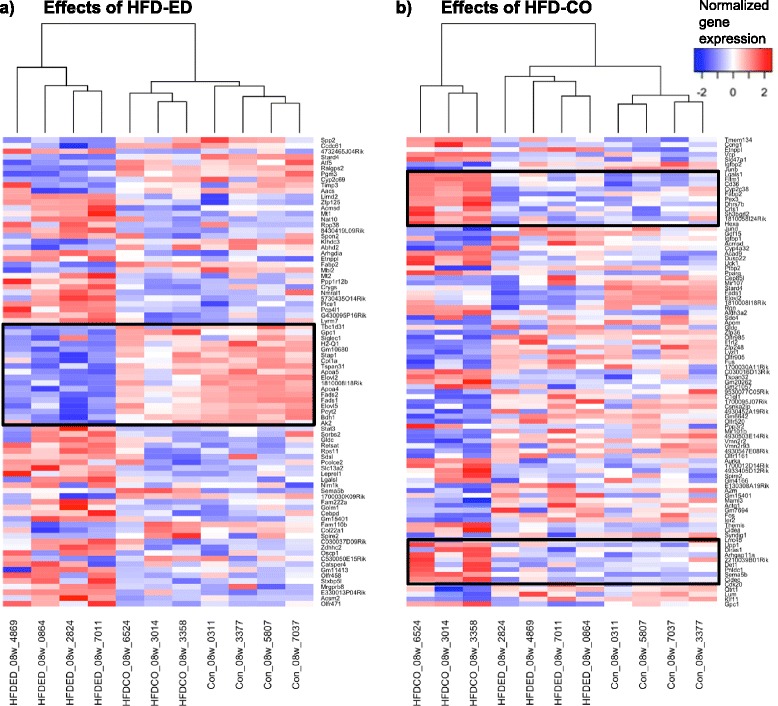
Heatmaps of individual gene expression that were regulated at 8 weeks by **a**) HFD-EPA/DHA and **b**) HFD-corn oil *vs*. the other two diets. Not annotated (“NA”) transcripts were excluded. The signals were normalised row wise to 0 and are shown from blue to red colour signifying the lowest to the highest gene expression values

Additional file 1: Figure S5a shows Venn diagram of the differentially expressed transcripts at 8 weeks; HFD-EPA/DHA *vs.* control (n = 1177), HFD-corn oil *vs.* control (n = 1288) and HFD-EPA/DHA *vs.* HFD-corn oil (n = 341). After 12 weeks, corresponding numbers were: HFD-EPA/DHA and control (n = 1370), HFD-corn oil *vs.* control (n = 1066) and HFD-EPA/DHA *vs.* HFD-corn oil (n = 142. The 89 transcripts in Additional file 1: Figure S5a that differed in both comparisons HFD-EPA/DHA *vs.* control and in HFD-EPA/DHA *vs.* HFD-corn oil at 8 weeks plus the 12 transcripts that differed between all three comparisons were further investigated (Fig. 3a). Corresponding gene expression data of the 61 transcripts at 12 weeks is shown in Additional file 1: Figure S6a, S6b and Fig. 3b show the 112 and 37 transcripts that were differentially regulated by HFD-corn oil compared to the other two diets at 8 and 12 weeks, respectively. These figures demonstrate that there is a clear time-dependence in that fewer genes were differentially regulated by either HFD at 12 weeks.

### Specific effects of HFD-EPA/DHA and of HFD-corn oil on gene expression

**Fig. 4:**
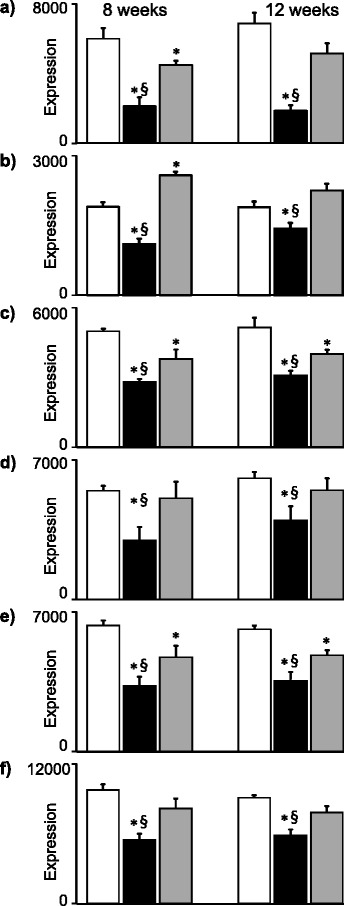
Expression of genes coding for proteins involved in lipid metabolism that were downregulated by HFD-EPA/DHA (black bars) *vs.* both control (white bars) and HFD-corn oil (grey bars). Results from 8 weeks (left panels) and 12 weeks (right panels) are shown. **a**) *ApoA4*. **b**) *Fabp2*. **c**) *Elovl2*. **d**) *Elovl5*. **e**) *Fads1* and **f**) *Fads2*. Gene expression (GE) from normalized microarray data and are shown in arbitrary units as mean ± SEM. FDR adjusted p-values p < 0.05; * *vs.* control. § *vs.* HFD-EPA/DHA

The boxed-in areas in the heatmap in Fig. 3a show genes that were downregulated by HFD-EPA/DHA compared with the other two diets at 8 weeks. Figure 4 shows the expression of some these genes at 8 weeks and 12 weeks. The genes are coding for proteins involved in lipoprotein particle assembly (*ApoA4*), fatty acid transport (*Fabp2*) and biosynthesis of long-chain PUFA (*Elovl2*, *Elovl5*, *Fads1* and *Fads2*). *ApoA5* was downregulated by HFD-EPA/DHA at 8 weeks but not at 12 weeks. Examples of genes that were upregulated by HFD-EPA/DHA compared with the other two diets were *Mt1* and *Mt2*.

**Fig. 5:**
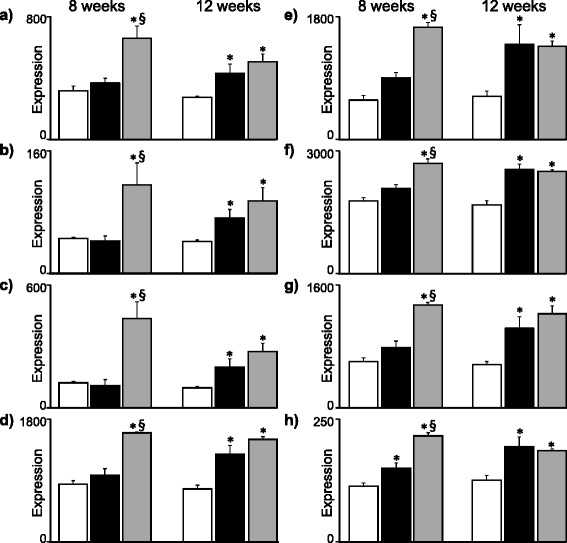
Expression genes coding for proteins involved in lipid droplet assembly or associated to lipid droplets in the cell upregulated by HFD-corn oil (grey bars) *vs.* both control (white bars) and HFD-EPA/DHA (black bars). Results from 8 weeks (left panels) and 12 weeks (right panels) are shown. **a**) *Pparg*. **b**) *Cidea*. **c**) *Cidec*. **d**) *Fitm1*. **e**) *Cd36*. **f**) *Aldh3a2*. **g**) *Lgals1* and **h**) *Sema5b*. Gene expression (GE) from normalized microarray data and are shown in arbitrary units as mean ± SEM. FDR adjusted p-values p < 0.05; * *vs.* control. § *vs.* HFD-EPA/DHA

The boxed-in areas in the heatmap in Fig. 3b show genes that were upregulated by HFD-corn oil compared with the other two diets at 8 weeks. Figure 5 shows expression of genes that were upregulated by HFD-corn oil *vs.* both control and HFD-EPA/DHA at 8 weeks but did not differ between the two HFDs at 12 weeks. These genes are known to be regulated by Pparγ, several of which are associated to cytoplasmic lipid droplet formation in hepatocytes (*Cidea*, *Cidec*, *Fitm1*, *Cd36*, *Aldh3a2*).

**Fig. 6:**
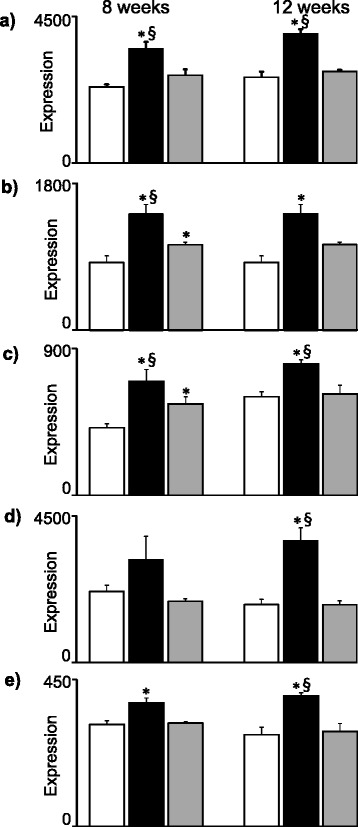
Expression genes upregulated by HFD-EPA/DHA *vs.* control and HFD-corn oil at 8 weeks (left) or at 12 weeks (right): **a**) *Retsat*. **b**) *Slc22a*. **c**) *Hsd17b6*. **d**) *Etnppl*. **e**) *Mreg*. Gene expression (GE) from normalized microarray data and are shown in arbitrary units as mean ± SEM. FDR adjusted p-values p < 0.05; * *vs.* control. § *vs.* HFD-corn oil

The final Fig. 6 shows gene pattern that were upregulated by HFD-EPA/DHA at 8 weeks *vs*. both control and HFD-corn oil, and with similar expression pattern at 12 weeks. These genes were *Retsat*, *Slc22a5*, *Hsd17b6*, *Etnppl* and *Mreg*.

## Discussion

The data presented in this study show that mice fed the EPA/DHA-enriched HFD were metabolically healthier than the HFD-corn oil mice despite similar degree of adiposity at 12 weeks. The metabolic health was expressed as the lower hepatic triglyceride levels in the mice fed HFD-EPA/DHA compared to the HFD-corn oil fed mice at both the time points. Transcriptomics analyses revealed that expression of genes coding for proteins associated to lipid droplets in hepatocytes were upregulated in the HFD-corn oil livers consistent with increased lipid storage. In contrast, Pparα-related gene expression was upregulated by the EPA/DHA-enriched diet suggestive of increased usage of lipids as fuel.

EPA and DHA enriched diets have been shown to reduced the risk of metabolic syndrome [10]. The fatty acid profile of diets shows that HFD-EPA/DHA diet provided a surplus of n-3 PUFAs since EPA and in particular DHA was present in the neutral lipid fraction. The mice fed the HFD-corn oil diet showed instead an excess of MUFA and n-6 PUFA storage in the hepatic triglycerides. Whether this difference in fatty acid profiles as a result of the two HFD is directly related to the much lower hepatic triglyceride content in the HFD-EPA/DHA mice than in the HFD-corn oil mice remains to be elucidated. Similar to our study, others have shown that dietary fish oil or EPA/DHA in HFDs counteracts lipid storage in livers of C57BL male mice [11–13]. One explanation to the lower liver triglyceride content after the HFD-EPA/DHA feeding could be upregulation of fatty acid β-oxidation since this is normally the major pathway to dispose excess fatty acid in the liver [2]. This is in line with the finding that C57BL mice fed HFD enriched in fish oil had lower level of hepatic steatosis and increased energy expenditure [14]. Alternatively, it has been shown that the n-6:n-3 ratio was increased in fatty livers from patients undergoing bariatric surgery [15]. This could be either caused by the increased n-6:n-3 ratio common in present day diets or the increased ratio could be a sign of inflammation in the liver, causing consumption of n-3-PUFA [16]. In the present study, the ratio of n-6:n-3 in the three diets were 1:62, 1:2.1 and 1:64 (control, HFD-EPA/DHA and HFD-corn oil, respectively). This may have bearing on as to why the triglyceride content at 8 weeks was lower in livers from the HFD-EPA/DHA mice even when compared to the controls.

The DXA-measurements performed at 12 weeks were consistent with the epiWAT and rpWAT relative weights. We can therefore assume that the HFD-EPA/DHA mice also had lower body fat % compared HFD-corn oil after 8 weeks diet intervention. This is in agreement with a previous study showing that male C57BL mice fed a high fat/high sucrose diet containing 5 wt.% EPA for 20 weeks had lower white adipose tissue depots weight than those fed the same diet but without EPA [17]. The dose of EPA and DHA used in this study, 0.83 wt.%, was much lower which may explain why the HFD-EPA/DHA mice in this study increased in adiposity after 12 weeks diet intervention. In addition, a lower hepatic triglyceride content has previously been shown to associate with lower epiWAT weight in C57BL mice fed HFD with EPA/DHA [12].

The family of Ppar transcription factors are widely believed to be primary mediators of the effects of EPA and DHA. These transcription factors regulate several lipid metabolic pathways in liver, adipose tissues and skeletal muscle. In obese humans with NAFLD, hepatic PPARG expression was upregulated [18]. Activation of Pparα reduces lipid storage in liver by promoting fatty acid β-oxidation [2]. In mice, Pparg overexpression induced hepatic steatosis whereas its disruption improved fatty liver [19]. The elevated *Pparg* gene expression at 8 weeks in the HFD-corn oil livers could directly relate to the increased lipid content detected in these livers. In contrast, there was no difference in hepatic *Pparg* gene expression in HFD-corn oil and HFD-EPA/DHA mice at 12 weeks. This could be an early sign of developing hepatic steatosis in HFD-EPA/DHA mice since Pparγ activation triggers a signalling cascade contributing to the formation of lipid droplets in the cytoplasm. *Cidec* (a.k.a. *Fsp27*), which was upregulated in the mice fed HFD-corn oil at 8 weeks, was regulated by Pparγ in steatotic livers from ob/ob mice [20]. However, in lean mice Cidec is regulated by Pparα, and recent research showed that simultaneous activation of Pparα and silencing of Cidec protected diet-induced obese mice from hepatic steatosis [21]. Other genes that are associated to LD formation, *Cidea* and *Fitm1* were also expressed at higher levels at 8 weeks in the HFD-corn oil livers compared to livers from the other two diets. Cidea is usually expressed at low levels in mouse liver but can be induced by treatment with Pparα agonists or by Pparγ overexpression [9]. Overexpression of *Fitm1* induced LD formation *in vivo* in mouse liver [22]. Cd36 is also regulated by Pparγ and, although not directly associated with LD, increased hepatic *Cd36* expression with age was recently shown to be associated with increased susceptibility to NAFLD [23]. The other gene transcripts such as *Aldh3a2*, *Lgals*, and *Sema5b* were also upregulated in HFD-corn oil mice as compared to HFD-EPA/DHA and control at 8 weeks. Aldh3a2 could also be involved in the formation of LD as Aldh3b2 was recently shown to be a LD-associated protein [24]. It appears therefore that induction of these genes, most likely through Pparγ, precedes the increased triglyceride storage in liver caused by a HFD. Interesting, LD proteins were suggested as drug targets for treatment of NAFLD [25].

The expression of the gene cluster including *RetSat* was upregulated by HFD-EPA/DHA *vs.* control and HFD-corn oil fed mice at one or both time-points, and had similar expression pattern at the other time-point. This cluster may therefore represent putative mechanisms to understand how EPA/DHA maintains lower hepatic triglyceride levels even as a component in a HFD. The cluster members have two obvious commonalities. The first is that, two of them are regulated by Pparα. In hepatocytes, *RetSat* (a.k.a. *Ppsig*) is directly regulated by Pparα [26], where it converts retinol to all-trans-13,14-dihydro-retinol [27]. *Slc22a5* is also known to be regulated by Pparα and is coding for a plasma membrane carnitine transporter, which is an essential metabolite required for mitochondrial β-oxidation of fatty acids [28]. However, there were no dietary effects on *Ppara* transcript levels at either time-point (data not shown). The other common theme is that two genes are associated to Vitamin A metabolism; *Retsat* and *Hsd17b6*. Similar to RetSat, Hsd17b6 is also involved in retinoid metabolism [29]. However, Ppars form heterodimers with the retinoic acid X receptor (Rxr) and 9-cis retinoic acid is the most potent ligand of Rxr, with 40 times the affinity to the receptor compared with trans-retinols [30]. Induction of *Retsat* may therefore result in modification of Ppar-Rxr signalling. Alternatively, a recent paper on bioinformatics analysis of public microarray data identified *Retsat* as a key component in disorders relating to insulin resistance in both mice and humans [31], a condition strongly associated to NAFLD. This suggests that Retsat *per se* is of high relevance in NAFLD. *Etnppl* and *Mreg* were other genes that showed similar pattern to that of *Retsat* but little is known about these two genes and their involvement in hepatic function. The human homolog of *Etnppl* catalyse the catabolism of phosphoethanolamine [32] and *Mreg* is involved in biogenesis of melanosomes, a lysosome-like organelle in retinal epithelial cells and melanocytes [33].

## Conclusion

The data presented here show that EPA and DHA delayed the development of liver dysfunction in mice despite the catch-up in adiposity at 12 weeks. Further, the quality of dietary fat affected transcriptional programs initiated by Ppars differently as reflected by the gene-set enrichment analyses. The corn oil-based HFD induced Pparγ gene signatures, the EPA/DHA-enriched HFD induced genes known to be regulated by Pparα. In addition, the EPA/DHA-enriched diet also induced *Retsat* expression that may affect the Ppar system through the regulation of agonists to the Rxr heterodimer.

## Methods

### Animals and study design

The Animal Ethics Committee at the University of Gothenburg, Sweden, approved the study (Dnr: 253–2009). Six-week old C57BL/6J male mice (Harlan Laboratories, Netherlands) were housed in temperature and humidity controlled environment with 12 h light/dark cycle at our local animal facility. The mice were caged 5–6 per groups with *ad-libitum* access to water and chow. The number of animals per diet in each study was 12 per group initially. There were no differences in body weights at the start of the experiments. The body weights were recorded weekly. The mice were nine and ten weeks old at the start of 8 and 12-week study, respectively. Two mice from the control group in the 8-week study were removed due to fighting. In the 12-week experiment, the HFD-EPA/DHA group started with 11 animals and tissues from two animals from each group were perfused with saline for histochemistry and therefore excluded in the analyses.

The food was removed at 6 am and the mice were killed on three consecutive days between 9 am and 12 am by lethal intraperitoneal injections of sodium pentobarbital. Animals were selected randomly and alternatively from two diet groups each day. Blood was collected from the heart followed by cervical dislocation. Plasma was obtained by centrifugation at 4 °C of EDTA whole blood (10 min; 10,000 x g). Tissues were dissected out, weighed and frozen in liquid nitrogen. Tissues and plasma were stored at −80 °C.

### Diet composition

The EPA and DHA-enriched oils (EPAX 1050 and EPAX 6015) were a gift from EPAX AS (Lysaker, Norway; now part of the FMC Corporation, Pennsylvania, USA). Table 1 shows the diet compositions; the control diet provided 24 Energy % (E%) of protein, 12 E% of fat and 65 E% of carbohydrates, whereas the two HFD contained 25 E% of protein, 32 E% of fat and 44 E% of carbohydrates. The diets were prepared by Lantmännen (Kimstad, Sweden) and were delivered in powdered form. Therefore, the powdered diet and water was rolled into balls to make the texture more appealing to the mice when the diet was changed three times per week.

### Body composition

Retroperitoneal and epididymal white adipose tissues (rpWAT and epiWAT, respectively) were dissected out and weighed in both the 8 and the 12-week diet intervention study.

At 12 weeks, the mice were anesthetized with isofluorane (Baxter, IL, USA) on the day of termination and body composition was measured by Dual energy X-ray Absorption (DXA) using a Lunar PIXImus densitometer (Lunar Corp, WI, USA) as previously described [34].

### Isolation of total RNA and microarray analysis

Four mice were selected from each diet group on the basis of body weight, plasma triglyceride and plasma cholesterol levels. Total RNA from the livers was purified using the RNeasy Lipid Tissue Mini kit (Qiagen Nordic, Sollentuna, Sweden) according to the manufacturer’s instruction. Total RNA concentrations were measured using the NanoDrop 2000c UV–vis Spectrophotometer (Thermo Fisher Scientific, Gothenburg, Sweden). The RNA quality of samples were evaluated using the RNA 6000 Nano LabChip for Agilent 2100 Bioanalyzer (Agilent Technologies, Gothenburg, Sweden).

The RNA was labelled and hybridized to GeneChip® Mouse Gene 2.0 ST arrays (Affymetrix, CA, USA) at the SCIBLU Genomics core facility (Swegene Centre for Integrative Biology at Lund University, Sweden). The raw data is deposited in Gene Expression Omnibus database under accession number GSE65370.

### Data acquisition and analysis

The CEL files were imported to the window based Affymetrix® Expression Console™ software 1.3 for background correction and signal estimation from microarrays. The microarray data was normalized applying the iter-Probe Logarithmic Intensity Error algorithm together with the quantile normalization and perfect match probe only methods (Fig. 6). Empirical Bayes method from the limma package was then applied to the signals to calculate moderated t- and F-statistics, log odds and differential expression for comparisons between diets. Based on hierarchical clustering and scatter plots, one microarray from the HFD-corn oil group at 8 weeks was removed from subsequent analyses (Additional file 1: Figures S2-S3). The Piano package was used for Gene Set Enrichment Analysis (GSEA) to identify the total number of genes regulated and the direction of regulation [35]. Gene Ontology (GO) terms were annotated to each probe-set after performing GSEA. The reporter algorithm was used to analyse the functional enrichment level of individual GO term [36]. Heatmaps were generated using the -log_10_p-values to visualize enriched GO biological processes (BPs) terms.

### Analysis of plasma and liver lipids

Plasma total triglyceride and cholesterol was determined enzymatically with Konelab Autoanalyser version 2.0 at the Clinical Chemistry department, Sahlgrenska University Hospital, Gothenburg, Sweden.

Approximately 0.3 g of the livers were weighed and freeze-dried overnight. The freeze-dried livers were weighed again followed by lipid extraction using the Folch method [37]. Briefly, 5 ml chloroform: methanol (2:1 v/v) was added to each sample and mixed followed by 15–20 min ultrasonication at 40 Hz (Branson 8510, Branson Ultrasonics Corp, CT, US). One ml of physiological saline was added, mixed and centrifuged at 1700 × g in swing-out buckets for 5 min. The lower organic phase was taken off and kept, while the remainder was re-extracted with 1.5 ml chloroform: methanol. The two extracted organic phases were combined and evaporated under a flow of nitrogen and redissolved in 5 ml isopropanol. The samples were stored at −80 ° C until analysis. Hepatic triglyceride content was measured in the total lipid fraction using enzyme assay obtained from Sigma-Aldrich.

### Lipid profiling of liver and diets

Lipid classes were separated using solid phase extraction (SPE) according to [38] Phospholipid (C17:0) and triglyceride (C19:0) standards were added from the beginning. Briefly, the aminopropyl solid phase extraction columns were activated with 4 ml of hexane and then loaded with 1 ml of extracted liver lipids samples. Neutral lipids were eluted with chloroform/isopropanol (2:1); free fatty acids were eluted using diethylether/2 % acetone and phospholipids were eluted with methanol and evaporated under N_2_ stream. The samples were then methylated with 1 ml 10 % acetyl chloride in methanol/ 1 ml toluene and incubated overnight. By adding 0.3 ml water and 4 ml petroleum ether two phases were separated and the upper phase was pipetted carefully, evaporated under nitrogen stream and redissolved in 250 μl isooctane [39].

For lipid analysis of diets approximately 100 mg of finely ground freeze-dried diet was used. Lipid extraction using Folch method [37], separation of lipid classes and methylation was performed as described above. The methylesters were separated by gas chromatography (Agilent 7890A, Santa Clara, CA) and detected with mass spectroscopy. Chemstation software (Agilent Technologies, Santa Clara, CA) was used for evaluation. The samples were separated on a VF-WAX (30mx0.25x0.25 um d_F_) column (J&W Scientific, Folsom, CA) and quantified by electron ionization with a 5975C inert XLK EI/CI MSD with a triple-axis detector (Agilent Technologies).

### Statistical analysis

Changes in body weights, body composition, adiposity and blood and hepatic lipid data were tested by ANOVA followed by Tukey-honest significant difference test (Tukey-HSD) post-hoc tests. A p-value <0.05 was considered statistically significant. All statistical and microarray data analysis were performed with the help of R Studio software (Version 0.98.953 – © 2009–2013 RStudio, Inc.) and required Bioconductor packages. For the microarray data false discovery rate (FDR) adjusted p-values were calculated and FDR adjusted p < 0.05 was considered significant. All data are presented as mean ± SEM.

## Additional file

Additional file 1: Figure S1. Box and Wisker plots of the normalized of hepatic transcriptome log2 values showing boxes of 25^th,^ 50^th^ and 75^th^ percentile and 10^th^ and 90^th^. **Figure S2.** Dendogram of the hepatic transcriptome array data by unsupervised clustering. The scale on the left vertical bar is based on ward distance: a) unsupervised clustering of all arrays. b) Distribution of all arrays by unsupervised clustering of the microarray data after removing array HFDCO_8w_5592. **Figure S3**. Scatterplots of all microarray data to visualize the variation between the samples within each diet group: a) control diet. b) HFD-EPA/DHA diet. c) HFD-corn oil diet and d) 8 week HFD-corn oil with data from one microarray excluded (5592). **Figure S4.** Heatmaps showing results obtained from gene set enrichment analysis (GSEA) of hepatic transcriptome data comparing both the HFDs against control at 8 and 12 week diet intervention. Not annotated (“NA”) transcripts were excluded. The signals were normalised row wise to 0 and are shown from blue to red colour signifying the lowest to the highest gene expression values. **Figure S5.** Venn diagrams of number of genes that were significantly (adjusted p-value < 0.05. FDR) changed irrespective of direction after a) 8 weeks and b) 12 weeks of diet intervention. **Figure S6**. Heatmaps of genes regulated by a) HFD-EPA/DHA or b) HFD-corn oil at 12 weeks.

## Abbreviations

Aldh3a2: Aldehyde dehydrogenase family 3, subfamily A2
ApoA: Apolipoprotein A
BPs: Biological processes
Cidea: Cell death-inducing DFFA-like effector a
Cidec: Cell death-inducing DFFA-like effector c
DHA: Docosahexaenoic acid
DXA: Dual energy X-ray Absorption
Elovl: Elongation of very long chain fatty acids
Etnppl: Ethanolamine phosphate phospholyase
EPA: Eicosapentaenoic acid
Fabp: Fatty acid binding protein
Fads: Fatty acid desaturase
FDR: False discovery rate
Fitm1: Fat storage-inducing transmembrane protein 1
GO: Gene Ontology
GPCRs: G-protein coupled receptors
GSEA: Gene Set Enrichment Analysis
HFD: High fat diet
Hsd17b6: Hydroxysteroid (17-beta) dehydrogenase 6
Lgals1: Lectin, galactose binding, soluble 1
Mreg: Melanoregulin
NAFLD: Non-alcoholic fatty liver disease
Ppar: Peroxisome proliferator-activated receptor
Retsat: Retinol saturase
Sema5b: Semaphorin 5b
Slc22a5: Solute carrier family 22, member 5
WAT: White adipose tissue
