# Supplementary figures and images for: Eicosapentaenoic and docosahexaenoic acid-enriched high fat diet delays the development of fatty liver in mice
Source: Lipids Health Dis. 2015 Jul 22;14:74. doi: 10.1186/s12944-015-0072-8 (PMC4509768; doi:10.1186/s12944-015-0072-8)

Figure S1

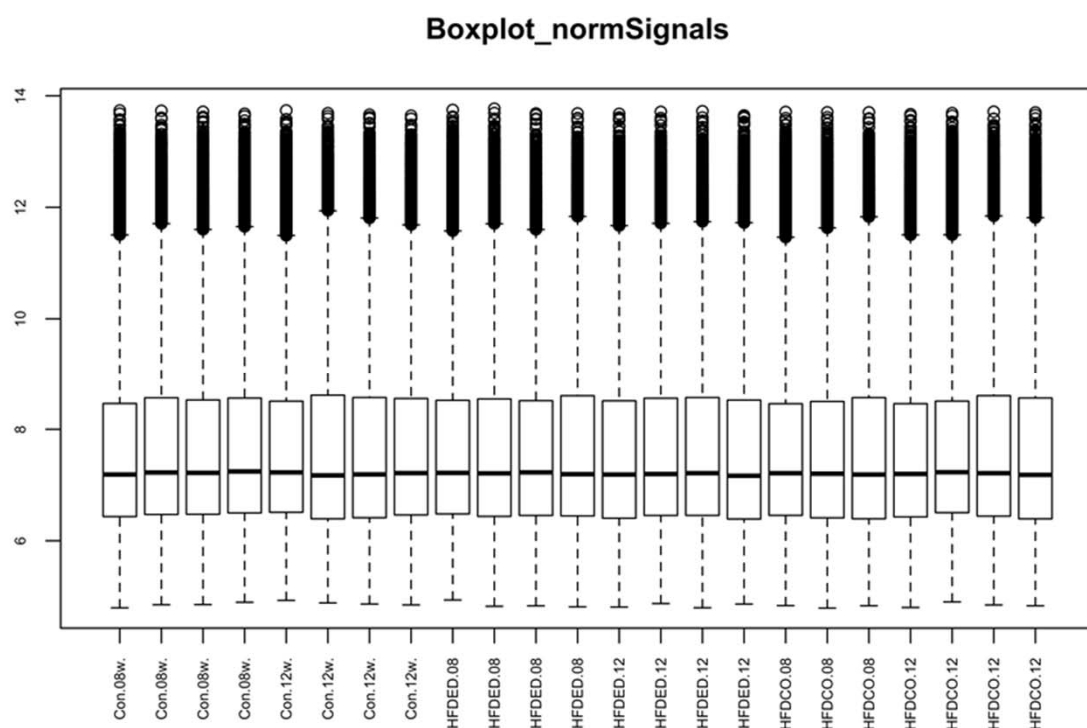

Figure S2

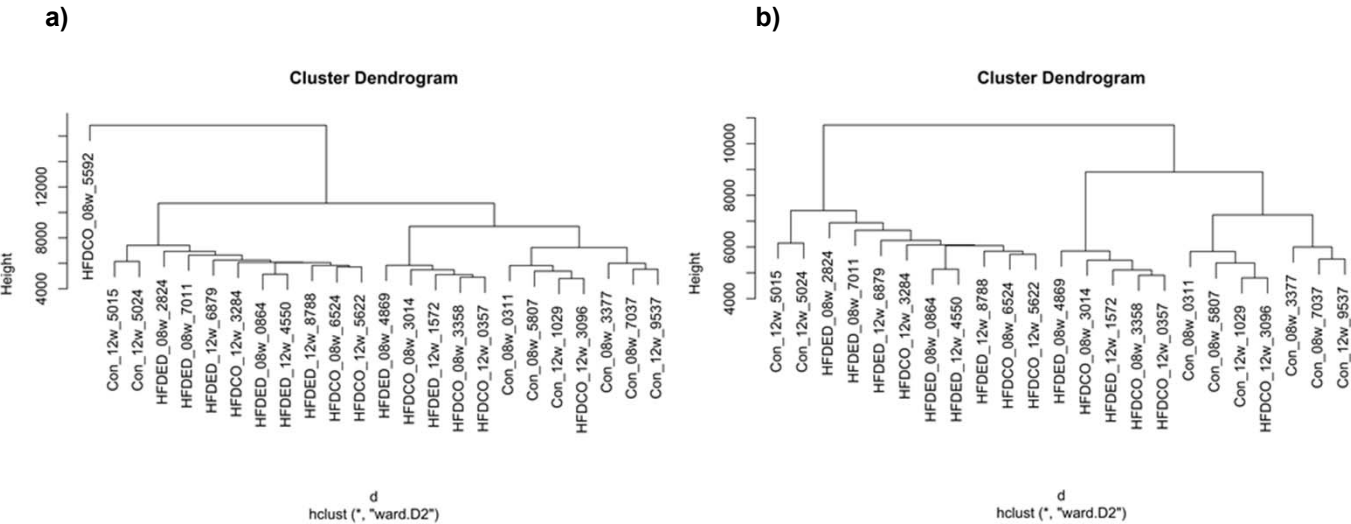

Figure S3

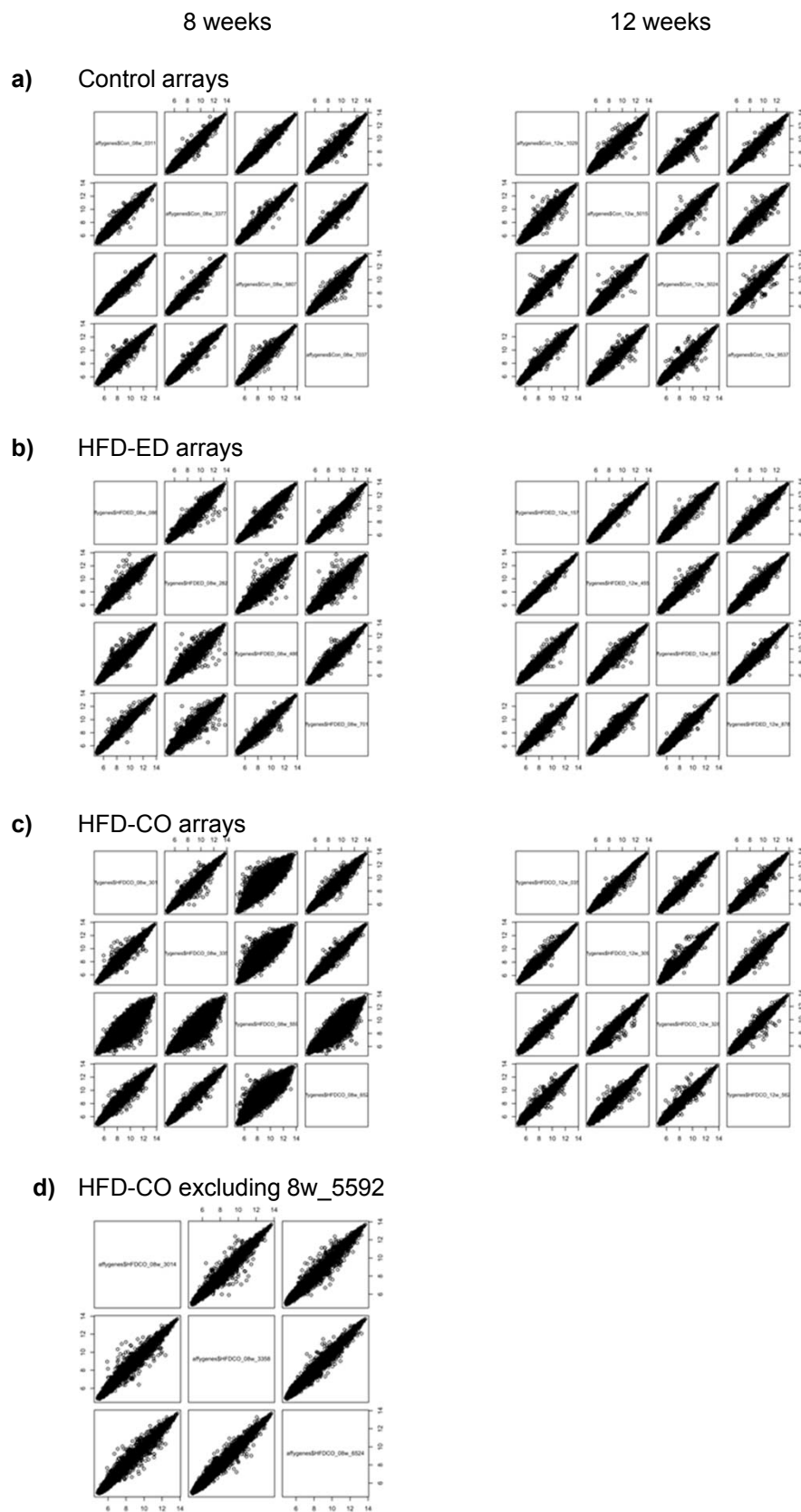

Figure S4

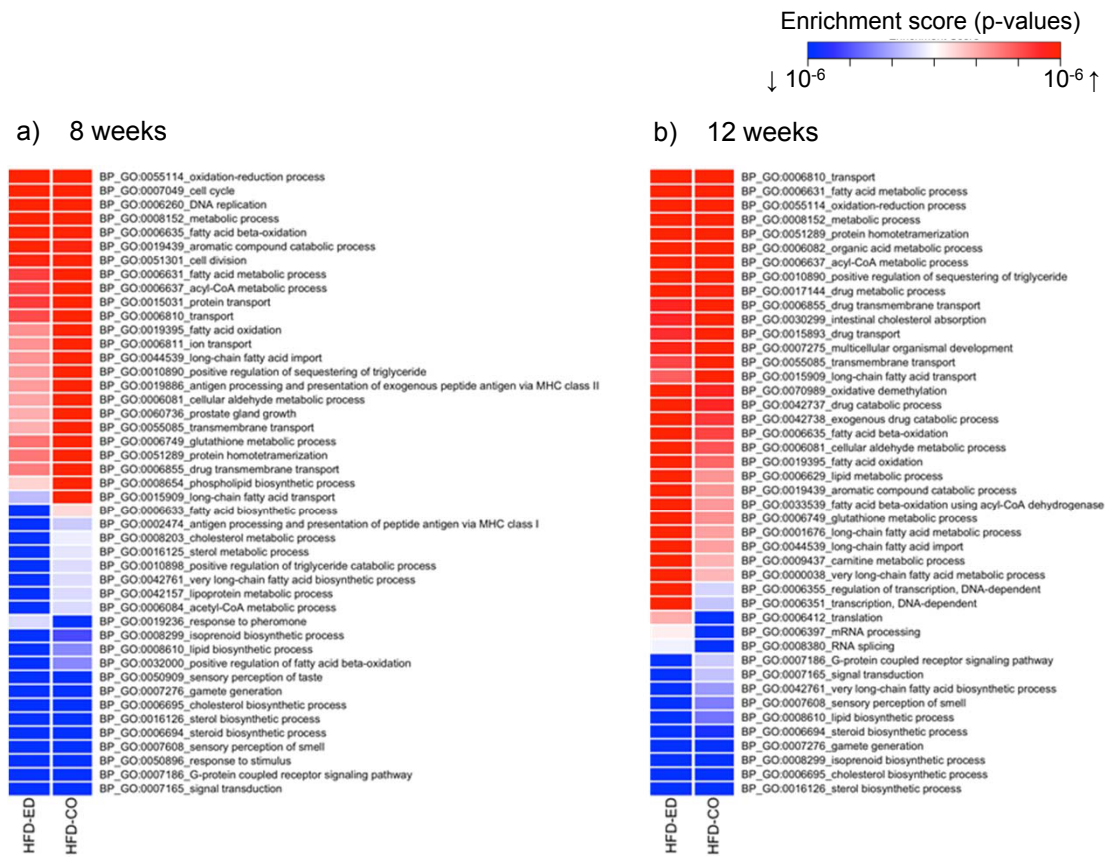

Figure S5

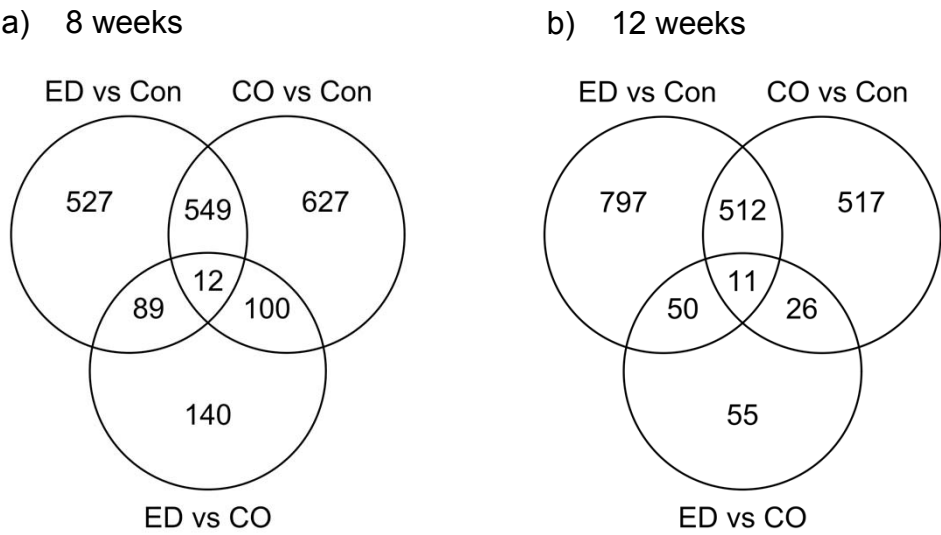

Figure S6

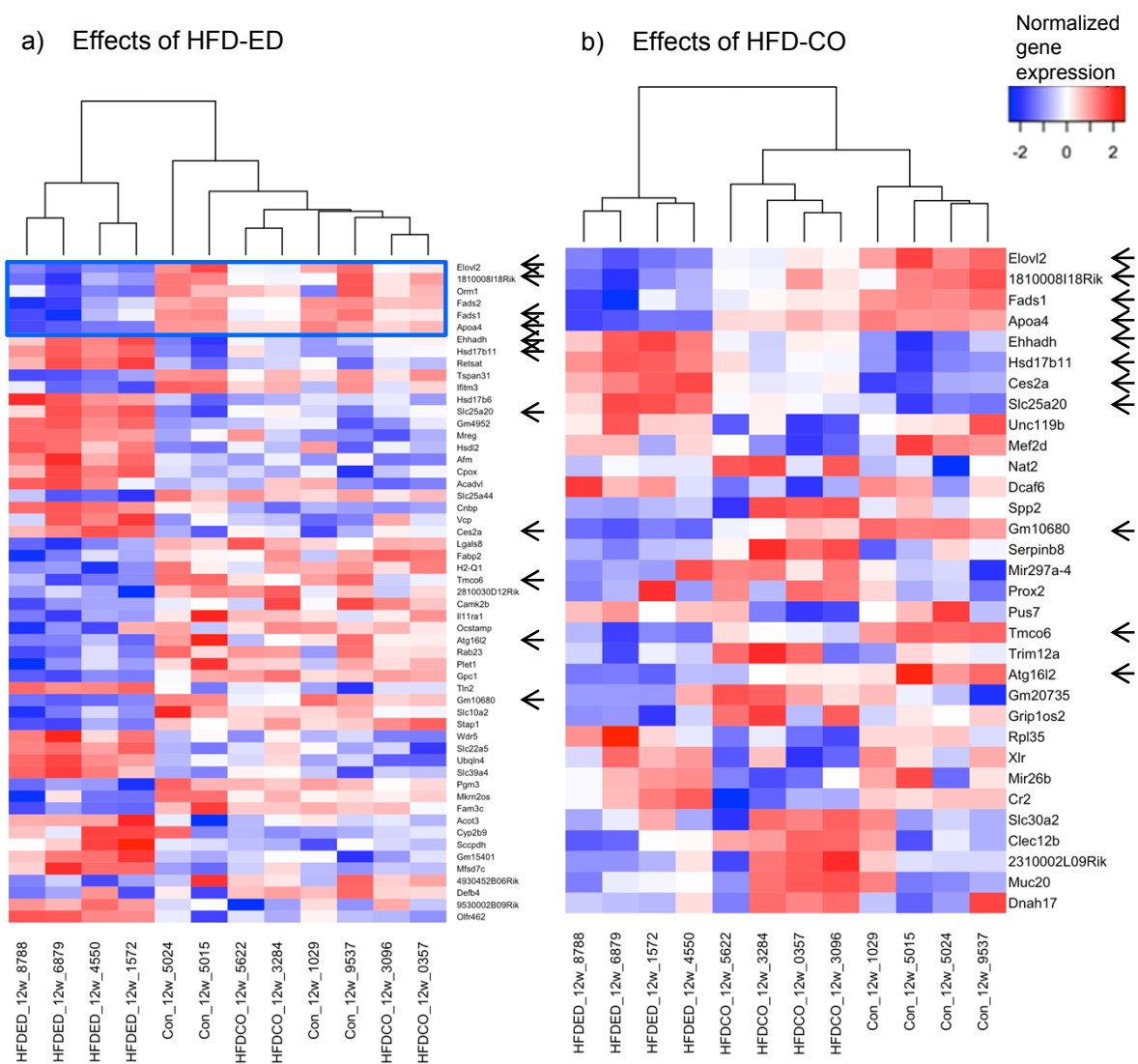

Supplement: Additional file 1: Figure S1. — Box and Wisker plots of the normalized of hepatic transcriptome log2 values showing boxes of 25th, 50th and 75th percentile and 10th and 90th. Figure S2. Dendogram of the hepatic transcriptome array data by unsupervised clustering. The scale on the left vertical bar is based on ward distance: a) unsupervised clustering of all arrays. b) Distribution of all arrays by unsupervised clustering of the microarray data after removing array HFDCO_8w_5592. Figure S3. Scatterplots of all microarray data to visualize the variation between the samples within each diet group: a) control diet. b) HFD-EPA/DHA diet. c) HFD-corn oil diet and d) 8 week HFD-corn oil with data from one microarray excluded (5592). Figure S4. Heatmaps showing results obtained from gene set enrichment analysis (GSEA) of hepatic transcriptome data comparing both the HFDs against control at 8 and 12 week diet intervention. Not annotated (“NA”) transcripts were excluded. The signals were normalised row wise to 0 and are shown from blue to red colour signifying the lowest to the highest gene expression values. Figure S5. Venn diagrams of number of genes that were significantly (adjusted p-value < 0.05. FDR) changed irrespective of direction after a) 8 weeks and b) 12 weeks of diet intervention. Figure S6. Heatmaps of genes regulated by a) HFD-EPA/DHA or b) HFD-corn oil at 12 weeks. [file 12944_2015_72_MOESM1_ESM.pdf]
